# Normalization of flow-mediated dilation to shear stress area under the curve eliminates the impact of variable hyperemic stimulus

**DOI:** 10.1186/1476-7120-6-44

**Published:** 2008-09-04

**Authors:** Jaume Padilla, Blair D Johnson, Sean C Newcomer, Daniel P Wilhite, Timothy D Mickleborough, Alyce D Fly, Kieren J Mather, Janet P Wallace

**Affiliations:** 1 Kinesiology, Indiana University, Bloomington, IN, USA; 2 Health and Kinesiology, Purdue University, West Lafayette, IN, USA; 3 Applied Health Science, Indiana University, Bloomington, IN, USA; 4 Medicine, Indiana University, Indianapolis, IN, USA

## Abstract

**Background:**

Normalization of brachial artery flow-mediated dilation (FMD) to individual shear stress area under the curve (peak FMD:SS_AUC _ratio) has recently been proposed as an approach to control for the large inter-subject variability in reactive hyperemia-induced shear stress; however, the adoption of this approach among researchers has been slow. The present study was designed to further examine the efficacy of FMD normalization to shear stress in reducing measurement variability.

**Methods:**

Five different magnitudes of reactive hyperemia-induced shear stress were applied to 20 healthy, physically active young adults (25.3 ± 0. 6 yrs; 10 men, 10 women) by manipulating forearm cuff occlusion duration: 1, 2, 3, 4, and 5 min, in a randomized order. A venous blood draw was performed for determination of baseline whole blood viscosity and hematocrit. The magnitude of occlusion-induced forearm ischemia was quantified by dual-wavelength near-infrared spectrometry (NIRS). Brachial artery diameters and velocities were obtained via high-resolution ultrasound. The SS_AUC _was individually calculated for the duration of time-to-peak dilation.

**Results:**

One-way repeated measures ANOVA demonstrated distinct magnitudes of occlusion-induced ischemia (volume and peak), hyperemic shear stress, and peak FMD responses (all p < 0.0001) across forearm occlusion durations. Differences in peak FMD were abolished when normalizing FMD to SS_AUC _(p = 0.785).

**Conclusion:**

Our data confirm that normalization of FMD to SS_AUC _eliminates the influences of variable shear stress and solidifies the utility of FMD:SS_AUC _ratio as an index of endothelial function.

## Background

As a "barometer" for cardiovascular health status, brachial artery flow-mediated dilation (FMD) provides a bioassay for *in vivo *endothelial function [1]. The evidence supporting the dependency of FMD on the endothelium is based on the observation that, after removal of the endothelial lining, arteries lose their ability to dilate in response to an increase in flow [2]. Furthermore, FMD is partially to totally abolished after intra-arterial administration of a NO synthase blocker (i.e. L-NMMA) [3,4]. Nitric oxide is an anti-atherosclerotic molecule, and decreased NO bioavailability is a hallmark of pro-atherogenic states [5]. The biological significance of FMD, the relative simplicity and the non-invasive nature of the technique has motivated the dramatic growth in FMD research over the past 15 years. The widespread adoption of this ultrasound technique has been supported in part by published data suggesting that FMD correlates with invasive measurements of endothelial function in the coronary arteries [6,7] and may predict future cardiovascular events [8,9]. However, data are also available indicating that reduced FMD has weak or no association with other cardiovascular risk factors [10,11], is a questionable marker of the presence and severity of coronary artery disease [12], fails to show a prognostic value [13], and has poor diagnostic accuracy for identifying older adults with subclinical cardiovascular disease [14]. These contradictory results may be due to substantial discrepancies in FMD protocols across labs, but also due to the inter-subject variability in hyperemic shear stress (the stimulus for FMD). Large variability in reactive hyperemia has been documented among individuals and populations [15]. The unadjusted FMD outcome may reflect conduit artery endothelial function as well as the magnitude of the hyperemic stimulus. Current work is moving to adjust the measured vasodilation response for the applied stimulus; in other words, dividing the peak FMD by the magnitude of stimulus achieved with reactive hyperemia. In 2005, Pyke and Tschakovsky [15], the initiators of this approach, suggested the utilization of shear rate (or shear stress (SS)) area under the curve (SS_AUC_) (individual area until peak FMD) as a method to quantify the entire and "relevant" hyperemic stimulus; and thus to be used for FMD normalization purposes. Two years later [16], the same authors provided experimental evidence of the physiological appropriateness of using this method. They concluded that the SS_AUC_, but not the peak shear, was the critical determinant of the peak FMD response. Following publication of this paper, a radical change in clinical practice within the FMD community was expected; however, to our surprise, since the time of Pyke and Tschakovsky's publication (April 2007), only two [17,18] out of 92 FMD studies (PubMed search) have incorporated normalization of FMD to SS_AUC_. Reasons for this slow adoption of the recently proposed method to express FMD data may include 1) insufficient lead time after publication of the article for dissemination and implementation; 2) inability to simultaneously capture arterial diameter and velocity; 3) lack of clinical evidence supporting the utility of this approach; and 4) complicated comparison with the large body of evidence collected using the traditional FMD approach.

A "proof of concept" study was conducted herein to further evaluate the efficacy of FMD normalization. In a group of healthy individuals, we elicited five different magnitudes of reactive hyperemia-induced shear stress to *mimic *real-life inter-population differences. Considering this analogy, we created the following experimental scenario: comparison of five hypothetical "populations" with different magnitudes of reactive hyperemia-induced shear stress stimuli. Because the five "populations" do in fact have identical endothelial function, the correct outcome should be no detectable differences. We hypothesized that peak FMD:SS_AUC _ratio (normalization approach), but not peak FMD (traditional approach), would be the same among the five "populations" with identical endothelial function. This observation would corroborate that normalization of FMD to SS_AUC _eliminates the influences of variable shear stress found among populations, and solidify the utility of FMD:SS_AUC _ratio as an index of endothelial function.

## Methods

### Subjects

Twenty healthy, physically active young adults (10 men, 10 women) volunteered for this study. All subjects were free of recognized cardiovascular, pulmonary and metabolic diseases, non-hypertensive (resting blood pressure < 140/80 mm Hg), non-obese (body mass index < 30 kg/m^2^), non-smokers, and had no family history of heart diseases. No subjects were taking medications with vaso-active effects, including contraceptives. All procedures were approved by Indiana University Committee for the Protection of Human Subjects. Written informed consent was obtained from each subject prior to participation in the study.

### Study procedures

Procedures of the study consisted of a screening session and a vascular testing session. The screening session included completion of a medical history/health habits questionnaire, measurements of height, weight, resting blood pressure, and a fasting venous blood draw to obtain total cholesterol, low density lipoprotein cholesterol, high density lipoprotein cholesterol, triglycerides, blood glucose, and high sensitivity C-reactive protein. In addition, all subjects were familiarized with the equipment/testing room to be used during the vascular session.

**Figure 1:**
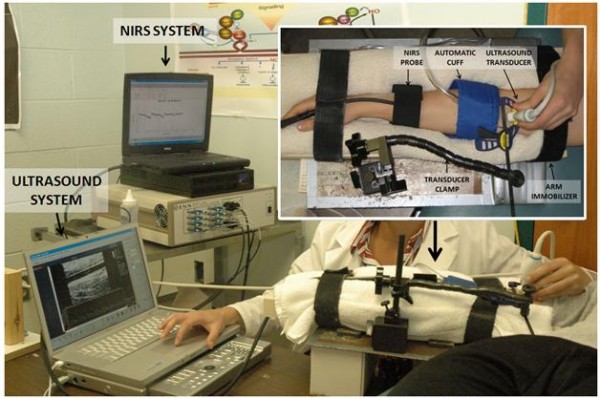
Experimental set-up.

On the vascular session day, subjects were instructed to report to the laboratory between 6:00 and 10:00 am having 1) fasted for 12 h, 2) abstained from caffeine, tobacco products, and vitamin supplements for 12 h, and 3) abstained from exercise for 12 h [1]. Women were studied during days 1–7 of their menstrual cycle to minimize the influence of cyclical changes in female hormones. Subjects were instructed to lie supine in a dark, climate-controlled quiet room (22–24°C), with their right arm extended out laterally. A venous blood draw was performed from the antecubital vein. Samples were collected into 6-mL heparin vacutainer tubes for determination of whole blood viscosity and hematocrit. Each subject underwent an acclimation phase (20 min) to obtain a hemodynamic steady state. Heart rate was continuously monitored using a three-lead ECG. Blood pressures were taken in the left arm with a mercury sphygmomanometer to confirm a steady state. A 5 × 84 cm automatic cuff (E-20 rapid cuff inflator, D.E. Hokanson, Bellevue, WA) was placed around the forearm immediately distal to the olecranon process following established guidelines for assessing FMD [1]. To quantify the magnitude of occlusion-induced ischemia (volume and peak), a dual-wavelength near-infrared spectrometry (NIRS) (ISS, Champaign, IL) probe was positioned over the right extensor digitorum muscle (medial aspect, distally from the cuff). The probe was held in place with an elastic bandage. The forearm oxygen tissue saturation (%StO_2_) was recorded throughout the study. The ultrasound image of the brachial artery was obtained longitudinally 2–10 cm above the antecubital fossa by 2D high resolution (Terason T3000, Teratech Corporation, Burlington, MA) ultrasound system, using a 5 to 12-MHz multifrequency linear-array transducer. Once a satisfactory image was obtained, the right arm was secured using a custom-designed arm immobilizer and the transducer was stabilized using a clamp (Figure 1). Minor corrections of transducer placement were made to maintain optimal imaging. Doppler velocity was also measured via ultrasound. Doppler flow signals were corrected at an insonation angle of 60° and measurements were performed with the sample volume placed in mid-artery. Ultrasound parameters were not changed during the study. Simultaneous Doppler measurements for blood velocity and 2D ultrasound imaging for diameter were continuously recorded for 30 sec at baseline. The automatic forearm cuff was then inflated to 250 mm Hg and maintained for 1, 2, 3, 4 or 5 min; in a randomized order. Diameter and velocity recordings resumed before cuff deflation and continued for 2 min thereafter. Ultrasound images were recorded at 5 frames/second using Camtasia (TechSmith, Okemos, MI) and converted into an AVI file. R-wave gated frames were not captured exclusively because internal data in our laboratory shows that continuous assessment of diameter at 5 frames/second yields the same FMD results (n = 10; FMD = 7.58 ± 0.9 vs. 7.62 ± 0.9%, p = 0.655; Intraclass correlation coefficient = 0.998, p < 0.0001). The occlusion conditions were applied 10 min apart from each other and baseline measurements were re-established prior to each condition.

### Data analysis

#### Brachial Artery Diameter and Blood Velocity

**Figure 2:**
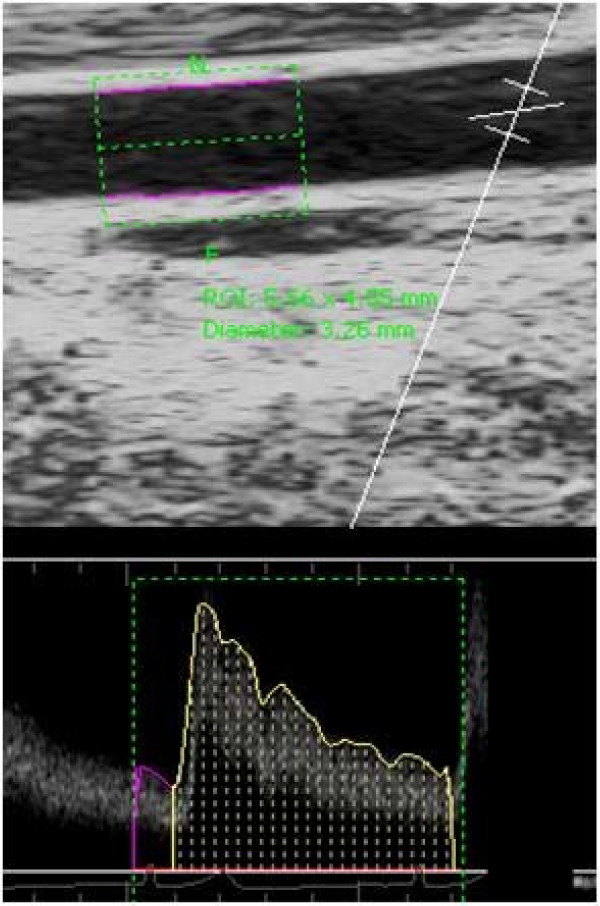
Analysis of brachial diameter and velocity (using the edge-detection Brachial Analyzer software) of a representative subject immediately following cuff deflation.

Off-line analyses of diameters and velocities were performed using an automated edge-detection Brachial Analyzer software (Medical Imaging Applications, LLC, Coralville, IA); briefly, the software allows the user to identify a region of interest (ROI) on the portion of the image where the vessel walls are most clear. The arterial wall borders were detected by an optimal graph search-based segmentation that uses a combination of pixel density and image gradient as an objective function. Each sequence of images was reviewed by the technician and interactively edited when needed to ensure that diameter measurements were always calculated from the intima-lumen interface at the distal and proximal vessel wall. Similarly, for determination of blood velocity, the ROI was selected around the Doppler waveform and the trace of the velocity-time integral was used to calculate mean velocity for each cardiac cycle (Figure 2). All measurements were performed by a single technician who was blinded to the trial condition for each image sequence. Reproducibility of our measurements has been reported previously [19]. The time course of diameters and velocities were determined using a 3-sec moving average. The peak dilation postocclusion was determined as the highest 3-sec average and was presented as a percent change from baseline diameter (peak FMD; %). Brachial artery blood flow was calculated using the following formula: Vm • *π *• (D^2^/4) 60, where Vm is mean blood velocity (cm • s^-1^), *π *is 3.14, and D is mean arterial diameter (cm) [20].

#### Whole blood viscosity

With the use of a pipette, 1 mL of blood sample was removed from the vacutainer tube, the mass of the sample was determined, and the density calculated. An additional blood sample was removed and transferred to a glass capillary viscometer [21] (Cannon-Manning Semi-Micro Viscometer, Cannon Instruments, Philadelphia, PA) and placed in a constant-temperature water bath (37°C). The viscosity of the sample was determined by measuring the time required for the sample to pass between two fixed points on the viscometer. Kinematic viscosity (mm^2 ^• s^-1^) was obtained by multiplying a viscometer constant (0.007927 mm^2 ^• s^-2^) by the efflux time in seconds. To obtain viscosity in mPa • s, kinematic viscosity was multiplied by the density in grams per milliliter. Measures were performed in duplicate.

#### Brachial artery shear stress

Brachial artery shear stress (dynes • cm^-2^) was calculated using the following formula: (4ηVm) • D^-1^, where η is blood viscosity (mPa • s), Vm is mean blood velocity (cm • s^-1^), and D is mean arterial diameter (cm) [22]. To describe the magnitude of reactive hyperemia-induced shear stress elicited with increased duration of occlusion, shear stress AUC was calculated for each occlusion condition. Briefly, the AUC was calculated by summing the areas of successive postocclusion trapezoids (each with a base of 3-sec) for 60 sec (SS60sec_AUC_; a.u.). To quantify the "relevant" hyperemic stimulus responsible for the peak FMD response, the shear stress AUC above baseline was individually calculated for the duration of time-to-peak dilation (SS_AUC_; a.u.). Normalization of FMD to shear stress was expressed as the peak FMD:SS_AUC _ratio (a.u).

#### Hematocrit

A 50–75 μL blood sample was removed from the vacutainer tube, transferred into a capillary tube and centrifuged for 5 min using a micro-hematocrit centrifuge (Clay-Adams, New York, NY). Hematocrit (%) was read using a micro-capillary reader (International Equipment Company, Needham Heights, MA). Measures were performed in duplicate.

#### Magnitude of ischemia

Forearm oxygen tissue saturation (StO_2_; %) was monitored at baseline (30 sec), throughout each occlusion period, and during the reperfusion phase. The time course of StO_2 _was determined using a 3-sec moving average. To quantify the volume of ischemia, the StO_2 _AUC (area below baseline) was calculated by summing the areas of successive trapezoids (each with a base of 3-sec) for the total duration of the occlusion period (StO_2AUC_; a.u.). Peak ischemia (StO_2peak_; %) was considered as the StO_2 _change from baseline to immediately before cuff release.

### Statistical analysis

Descriptive statistics were used to summarize the subject demographic data. One-way repeated measures ANOVA (5 levels) were performed to determine the effect of forearm occlusion duration on volume of ischemia (StO_2AUC_; a.u.), peak ischemia (StO_2peak_; a.u.), magnitude of reactive hyperemia-induced shear stress (SS60sec_AUC_; a.u.), peak FMD response (%), and FMD normalized to shear stress (FMD:SS_AUC _ratio; a.u.). Tukey's HSD procedure was used when a significant F-ratio was found. All data are presented as mean ± standard error of the mean (SEM). For all statistical tests, the alpha level was set at 0.05. Statistical analyses were performed with SPSS v.15.0. (SPSS, Inc. Chicago, IL, USA).

## Results

**Figure 3:**
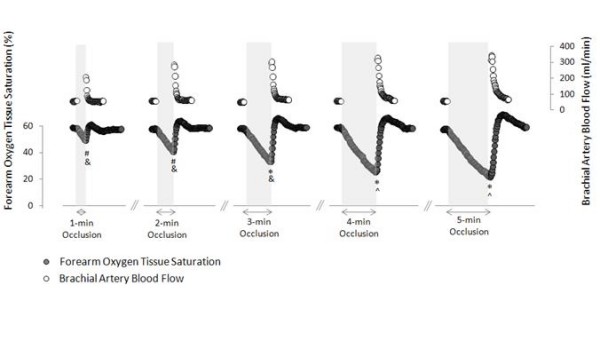
**Time course of forearm oxygen tissue saturation (NIRS) in conjunction with brachial artery blood flow (ultrasound)**. Values are means. *Volume of ischemia (StO_2AUC_) significantly different from all other trials; ^#^Volume of ischemia (StO_2AUC_) significantly different from 3-min, 4-min, and 5-min; ^&^Peak ischemia (StO_2peak_) significantly different from all other trials; ^^ ^Peak ischemia (StO_2peak_) significantly different from 1-min, 2-min, and 3-min. All p < 0.0001. Ultrasound data were not collected during the occlusion periods.

**Figure 4:**
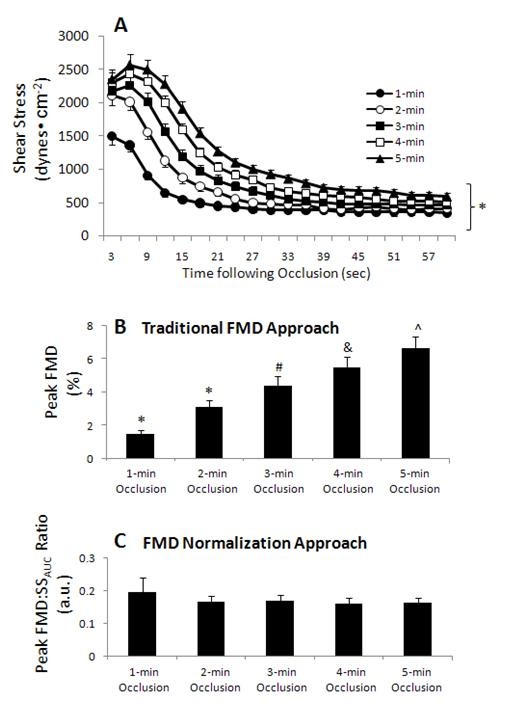
**Reactive hyperemia-induced shear stress (panel A), peak FMD response (panel B), and FMD normalized to shear stress (panel C) for the five occlusion conditions**. Values are means ± SEM. Panel A: *Magnitude of reactive hyperemia-induced shear stress (SS60sec_AUC_) significantly different among all trials. Panel B: *Peak FMD significantly different from all other trials; ^#^Peak FMD significantly different from 1-min, 2-min, and 5-min; ^&^Peak FMD significantly different from 1-min and 2-min; ^^^Peak FMD significantly different from 1-min, 2-min, and 3-min. All p < 0.0001.

Demographic information of the subjects is summarized in Table 1. No effect of sex was found in any of the main outcome variables (SS60sec_AUC_, peak FMD response, FMD:SS_AUC _ratio), thus data were pooled across sex. Table 2 displays mean baseline values for brachial artery diameter, blood velocity, shear stress, heart rate and forearm oxygen tissue saturation. No differences in baseline values were found (p > 0.05) for any of the variables. NIRS data on one subject was lost, thus sample size was 19 for all NIRS-related analysis. Figure 3 illustrates the time course of forearm oxygen tissue saturation in conjunction with brachial artery blood flow across the different forearm occlusion conditions. As indicated, the volume of ischemia and peak ischemia increased incrementally with duration of forearm occlusion (F_(4,72) _= 102.6; p < 0.0001 and F_(4,72) _= 69.85; p < 0.0001, respectively). Figure 4 illustrates the effects of forearm occlusion duration on the magnitude of reactive hyperemia-induced shear stress (panel A), peak FMD response (panel B), and FMD normalized to shear stress (panel C). As shown, varying forearm occlusion duration effectively elicited five different magnitudes of reactive hyperemia-induced shear stress (F_(4,76) _= 97.6; p < 0.0001). As a result, differences in peak FMD response were detected among conditions (F_(4,76) _= 40.5; p < 0.0001); however, these discrepancies were abolished when normalizing FMD to the shear stress (peak FMD:SS_AUC _ratio) (F_(4,76) _= 0.43; p = 0.785).

**Table 1:** Demographic characteristics of the subjects.

Variable	Value
N	20
Sex, M/W	10/10
Age, years	25.3 ± 0. 6
Height, cm	175.0 ± 2.1
Weight, kg	68.6 ± 2.5
Body mass index, Kg/m^2^	22.3 ± 0.4
Resting systolic blood pressure, mmHg	112.0 ± 2.6
Resting diastolic blood pressure, mmHg	71.4 ± 2.2
Total cholesterol, mg/dL	159.3 ± 7.2
High density lipoprotein cholesterol, mg/dL	57.2 ± 2.4
Low density lipoprotein cholesterol, mg/dL	89.1 ± 6.2
Triglycerides, mg/dL	64.9 ± 5.4
Fasting glucose, mg/dL	90.3 ± 1.5
High sensitivity C-reactive protein, mg/L	0.50 ± 0.1
Whole blood viscosity, mPa·s	3.74 ± 0.1
Hematocrit, %	42.9 ± 0.8
Reported physical activity^1^, days/week	4.3 ± 0.2

Values are mean ± SEM.

^1 ^Aerobic physical activity of moderate intensity for 30 min or more.

**Table 2:** Baseline hemodynamic information among forearm occlusion durations.

Variable	1-min	2-min	3-min	4-min	5-min	*p *value
Arterial diameter, cm	0.352 ± 0.01	0.349 ± 0.01	0.349 ± 0.01	0.350 ± 0.01	0.350 ± 0.01	0.308
Blood velocity, cm • s^-1^	8.48 ± 1.9	8.99 ± 2.0	8.18 ± 1.8	8.81 ± 2.0	9.00 ± 2.0	0.67
Shear stress, dynes • cm^-2^	367.0 ± 32	392.4 ± 42	359.9 ± 32	385.1 ± 42	393.7 ± 40	0.648
Heart rate, bpm	51.5 ± 2	51.1 ± 2	52.5 ± 2	52.6 ± 2	52.4 ± 2	0.412
Forearm oxygen tissue saturation, %	56.87 ± 2.4	58.16 ± 2.3	58.46 ± 2.5	57.98 ± 2.5	58.41 ± 2.5	0.974

Values are means ± SEM. P value indicates the one-way ANOVA comparing the five baseline periods.

## Discussion

Given that reactive hyperemia varies among individuals and populations [15], the FMD outcome is reflective of both conduit artery endothelial function and magnitude of the hyperemic stimulus. Normalization of FMD to SS_AUC _has recently been proposed [16] to control for the presence of the large inter-subject variability in reactive hyperemia-induced shear stress and solely reflect conduit artery endothelial function. The present study was designed to further examine the efficacy of FMD normalization. In a group of healthy individuals, via manipulation of duration of cuff occlusion, we evoked five different magnitudes of reactive hyperemia-induced shear stress (Figure 4A) to create an idealized experimental scenario: comparison of five hypothetical "populations" with known identical endothelial function but different magnitude of reactive hyperemia-induced shear stress stimuli. Our findings demonstrate that, when presenting the data using the traditional approach (peak FMD), differences in FMD are detected across the varying shear stimuli (Figure 4B), which would suggest the erroneous conclusion that discrepancies in endothelial function exist among these populations. However, when presenting the data using the normalization approach (FMD:SS_AUC _ratio), these differences are abolished (Figure 4C); thus confirming that normalization of FMD to SS_AUC _eliminates the influences of variable shear stress on measured conduit artery endothelial function.

Pyke and Tshakovsky [16] were the first to examine the effect of normalizing FMD to SS_AUC_. The purpose of their study was to investigate the independent contributions of the peak and continued reactive hyperemia-induced shear stress on FMD. This was elegantly conducted by maintaining the peak shear stimulus constant while manipulating the shear stimulus duration (re-inflation of the cuff) or by maintaining the shear stimulus constant while manipulating the magnitude of the peak shear stimulus (application of arterial pressure). From these experiments, the authors concluded that the SS_AUC_, but not the peak shear, was the critical determinant of the peak FMD response; and thus to be used for normalization purposes. Because their findings were convincing and physiologically sound, we did not consider further exploration of the relative contribution of peak vs continued shear. Instead, we opted to manipulate the duration of cuff occlusion because this is an effective method to globally modify the hyperemic stimulus (both peak and duration); thus closely mimicking real-life between-subject differences in shear.

The only two ultrasound studies manipulating the duration of cuff occlusion as strategy to create a range in hyperemic stimuli and assess the subsequent vasodilation response at the conduit artery (radial and brachial) were published in 1997 [23,24]. Both groups concluded that the duration of hyperemic stimulus played an important role on the vasodilatory response, observations that prompted Pyke and Tshakovsky's study [16] 10 years later. Our data are in agreement with Leeson et al. [24] and Joannides et al. [23] in that longer duration of cuff occlusion was associated with greater hyperemic and vasodilation responses; however, these groups did not test whether normalization of FMD to SS_AUC _removed differences among trials; this is not surprising as the concept of FMD normalization was not introduced at that time. A limitation to the studies of Leeson et al. and Joannides et al. is the utilization of blood flow, instead of shear stress, as representation of the vasodilatory stimulus. Unfortunately, blood flow (velocity • *π *• (diameter^2^/4) • 60) and shear stress (viscosity • 4 • velocity/diameter) are parallel only in conditions where arterial diameters are constant, a situation that does not occur during the hyperemic phase where arterial diameters are significantly and dynamically altered. Furthermore, in contrast to Leeson et al. and Joannides et al., our study incorporated the NIRS technology to quantify the magnitude (volume and peak) of forearm occlusion-induced ischemia. Given that the forearm ischemia is the driving force for reactive hyperemia, characterization of the principal stimulus for FMD should not be neglected.

There are a few limitations to this study. First, because all trials were performed on the same day, it is possible that repeated episodes of ischemia-induced hyperemia influenced subsequent measures; however evidence suggests that serial FMD measurements do not affect subsequent FMD outcomes [19]. We chose to include all trials on one day in order to avoid day-to-day endothelial function variability [25] and to allow for consistent placement of the ultrasound and NIRS probes across trials, which minimizes these recognized sources of variability in these measurements. Sufficient time was allowed between trials for diameters, velocities, and tissue oxygen saturation to reach baseline values. Most importantly, the order of trials was randomized to minimize any confounding influence from this potential carry-over effect. Second, although the dilation following the conventional 5-min forearm occlusion is largely attributed to NO[3,4], it remains unknown if dilation following shorter periods of occlusion (1, 2, 3, and 4 min) is NO dependent as well. In addition, the effects of reduced shear and/or metabolites released during the ischemic period on the FMD response are not well understood, thus manipulation of occlusion duration may be considered a limitation to the study. Third, our measurements of whole blood viscosity were performed using a glass capillary viscometer, thus we were unable to assess viscosity at varying shear rates. Although we believed that measurements of viscosity using this method would provide more accurate shear stress information than using a constant (assumed) viscosity of 4 mPa • s for all subjects [26], there were no differences between both approaches (Table 3). This observation suggests that the addition of viscosity measurements does not have a significant impact on shear stress calculations and it does not alter the interpretation of the FMD results. The validity of our viscosity measures may be demonstrated by the small coefficient of variation (4.47 ± 0.65%) and high correlation between hematocrit and viscosity (r = 0.878, p < 0.001). Fourth, because our sample was composed of 20 healthy asymptomatic men and women, extrapolation of these findings across clinical populations should be made with caution.

**Table 3:** Shear stress area under the curve calculated using measured viscosity vs. assumed viscosity.

	Shear stress area under the curve (a.u.)			
			
	Measured viscosity used	Assumed viscosity (4 mPa·s) used	t-test p value	ICC coefficient (p value)
1-min	11.7 ± 1.3	12.7 ± 1.5	0.610	0.985 (< 0.0001)
2-min	20.0 ± 1.4	21.7 ± 1.7	0.457	0.950 (< 0.0001)
3-min	26.3 ± 1.6	28.7 ± 2.2	0.386	0.941 (< 0.0001)
4-min	35.4 ± 2.1	38.3 ± 2.5	0.377	0.930 (< 0.0001)
5-min	41.5 ± 2.2	45.0 ± 2.8	0.340	0.919 (< 0.0001)

Values are means ± SEM.

ICC = Intraclass correlation

## Conclusion

The significance of the present findings is notable. Our results support the view of Pyke and Tschakovsky [16], the original advocates of FMD normalization to SS_AUC _approach. The obvious lack of compliance to the recent guidelines in the clinical literature is intriguing and perhaps problematic. From a theoretical and physiological standpoint, there is robust evidence that FMD should be corrected by its dilator stimulus; however, the following question remains to be resolved: Does FMD:SS_AUC _ratio predict cardiovascular risk and events more accurately than peak FMD (the traditional FMD approach)? Further research is warranted to confirm that the apparent physiological appropriateness of normalization is in fact clinically pertinent. Given that arterial diameter and velocity measurements are typically performed continuously and simultaneously during the hyperemic phase, we encourage investigators to, at the minimum, report FMD data using both the traditional (peak FMD response) and normalization (peak FMD:SS_AUC _ratio) approaches. In supporting this new approach, while we do not intend to invalidate the large body of evidence collected using the traditional FMD approach, we advise the reader to interpret the existing literature with caution. Special attention should be devoted to studies that did not report any form of hyperemic stimulus.

## Competing interests

The authors declare that they have no competing interests.

## Authors' contributions

JP designed the study, recruited the subjects, collected the data, analyzed the data, and wrote the original manuscript draft. BDJ and DPW assisted in data collection and edited the manuscript draft. SCN, TDM, ADF and KJM assisted in study design and edited the manuscript draft. JPW assisted in study design, supervised the study, and edited the manuscript draft. All authors read and approved the manuscript.
